# Predicting Thermodynamic Stability at Protein G Sites with Deleterious Mutations Using λ-Dynamics with Competitive Screening

**DOI:** 10.1021/acs.jpclett.5c00260

**Published:** 2025-03-21

**Authors:** Christopher Yeh, Ryan L. Hayes

**Affiliations:** † Department of Pharmaceutical Sciences, University of California Irvine, Irvine, California 92697-3958, United States; ‡ Department of Chemical and Biomolecular Engineering, University of California Irvine, Irvine, California 92697-2580, United States

## Abstract

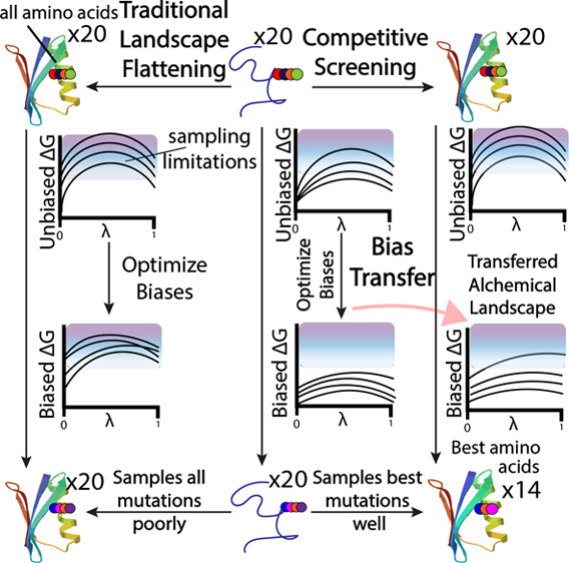

Free energy predictions are useful in protein design and computer-aided drug design. Alchemical free energy methods are highly accurate, and the alchemical method λ-dynamics significantly improves computational cost. Recent progress made simulations of dozens of perturbations at a single site possible, enabling *in silico* site-saturation mutagenesis with λ-dynamics. Site-saturation mutagenesis may require increased sampling to characterize many mutations and to accommodate structural disruptions around deleterious mutations. We reintroduce the neglected idea of competitive screening with λ-dynamics to address both issues. Traditional landscape flattening tunes two distinct biases to sample all mutations equally in the folded and unfolded states. Competitive screening transfers the unfolded bias to the folded state so that only reasonable mutations are sampled. Competitive screening is demonstrated on four surface sites and four buried sites in protein G and provides improvements for buried sites. Consequently, competitive screening provides new opportunities for molecular design within larger chemical spaces.

Alchemical free energy methods use molecular dynamics simulations to gain insight into a variety of biological processes, including solvation, binding, protonation, and molecular stability.^1,2^ These predictions can be used in applications such as computer-aided drug design (CADD) and protein engineering.^3−6^ Alchemical methods evaluate the relative free energy difference between related chemical systems via rapidly converging alchemical processes instead of slowly converging physical processes such as ligand binding and protein unfolding. Alchemical methods typically utilize a coupling parameter λ introduced into the potential energy function such that λ = 0 and λ = 1 represent two distinct chemical states, and the values of λ between those end-states represent nonphysical alchemical intermediates. Free energy calculations using current approaches suggest that computed free energies are generally accurate to experimentally determined free energies to within 0.5–1.5 kcal/mol, which allows candidate designs to be suggested for experimental verification.^3,6−9^

Previous studies on protein stability and CADD have employed both traditional alchemical methods such as free energy perturbation and newer nonequilibrium methods.^5,6,10,11^ However, these methods are limited by high computational cost and poor scaling. First, traditional methods break the alchemical transformation into many steps along λ, which each require their own simulation. Second, these methods can only make pairwise comparisons between two end-states in one set of simulations; thus, the computational cost scales linearly with the total number of chemistries, either ligand perturbations or sequence mutations. For comparisons between large sets of ligands the computational cost quickly increases to unfeasible levels, so methods that reduce the computational cost from these pairwise approaches for multiple chemistries are desirable to improve the efficiency of free energy calculations.

λ-dynamics is an efficient and scalable alchemical free energy method that addresses these issues.^12^ Instead of the discrete states used in traditional alchemical free energy methods, the coupling parameter λ is allowed to fluctuate in value analogously to the spatial coordinates of the system. The ability to fluctuate between λ values allows for the sampling of many chemical groups in a single simulation by generalizing from a single dimensional λ variable to a multidimensional λ space. This expansion of chemical space allows efficient quantification of free energies for many chemical systems in a single simulation, which is many times more efficient than the linearly scaling pairwise comparisons needed in traditional free energy methods. Traditional free energy methods may require less sampling to evaluate protein thermostability for a single mutant, but many pairwise comparisons between many mutants scales to be more computationally expensive than a single intensive λ-dynamics simulation of all mutants.

In recent years, many developments have enabled λ-dynamics to more efficiently explore chemical space, beginning with the multisite generalization to explore combinatorial spaces in a single simulation.^13^ Next, sampling improvements were achieved through the development of implicit constraints, which focused sampling away from alchemical intermediates, and the development of enhanced sampling algorithms to accelerate alchemical transitions with adaptive landscape flattening (ALF) and biasing potential replica exchange.^14−16^ The soft limitation of 8–9 substituents per site was overcome recently through the adoption of new implicit constraint bias terms and the nonlinear loss function in ALF, which allows efficient sampling of dozens of substituents per site.^17^ This new capability of λ-dynamics to sample dozens of substituents has significant implications for protein design because it enables calculating the thermodynamic stability of all 20 mutations of a protein residue simultaneously.

An accurate and efficient method to predict the thermodynamic stability of all protein mutants at a residue would be of great use in protein engineering, allowing computational design of new proteins or of enriched libraries for experimental screening. Thermodynamic stability is especially important as it impacts numerous protein characteristics such as structure, function, expression, and solubility. The ability of λ-dynamics to evaluate all amino acid mutations at a single residue in a single simulation makes it uniquely suited among free energy methods to design for stability within large protein design sequence spaces. However, sampling difficulties can arise with the large chemical space accessible by λ-dynamics when most mutations are deleterious. Specifically, destabilizing mutations can lead to slow structural rearrangements or partial unfolding, which cause kinetic artifacts on computationally accessible time scales if the mutations are sampled.

This work evaluates the efficiency and accuracy of λ-dynamics when calculating the thermodynamic stability within large mutation spaces at sites including many energetically unfavorable mutants, and introduces techniques to mitigate the associated sampling difficulties. The B1 domain of protein G was chosen for investigation as it has available crystal structures,^18^ and two experimental studies have measured the unfolding free energy Δ*G*_expt_ of every point mutation in protein G.^19,20^ These studies identified several sites in the hydrophobic core of the protein where the majority of mutations from the native residue were extremely thermodynamically destabilizing, leading to protein unfolding. Accurately characterizing unfavorable mutations at these constrained residues would require massive amounts of sampling to capture partial unfolding, significantly impeding convergence. A potential solution to these sampling difficulties is to tune the alchemical biases to prevent these destabilizing mutations from being sampled during simulation despite their inclusion in the ensemble. Then the remaining residues can be accurately characterized. Toward this end we reintroduce the idea of competitive screening in this letter.

In traditional landscape flattening (TLF), separate biases would be trained on both arms of the alchemical cycle to facilitate equal sampling of all mutations in each ensemble, which could allow very destabilizing mutations to disrupt the folded ensemble. Alternatively, earlier λ-dynamics studies described a competitive screening (CS) approach that acted as a competitive binding assay, where favorable states would be sampled more.^12,21^ These studies used reference energy values as the slope of a primitive linear bias to bias sampling toward the end states whose free energy was furthest below that reference energy. While the bias potentials of these studies were quite primitive, we can apply the idea of biasing toward more favorable mutations to the more developed bias potentials trained by ALF. In the context of ALF, this entails using ALF to flatten the alchemical landscape of the reference ensemble (for proteins, the unfolded peptide). The biases trained on the reference ensemble are then transferred to the target ensemble (the folded protein). Applying the unfolded ensemble biases to the folded ensemble automatically biases sampling toward mutants that are more favorable in the folded ensemble than in the reference ensemble. Therefore, CS primarily samples the most stabilizing mutations. Using this new iteration of CS with λ-dynamics, we can evaluate all reasonable mutations of a residue through free energy calculations more efficiently than before.

To evaluate the relative accuracy and precision of TLF and CS, several core and surface sites were selected. Mutations at the surface sites G9, T16, A20, and N37 had no large penalties on folding, while the majority of mutations at the core sites A26, F30, F52, and V54 were deleterious (Figure 1). The simulations were run using the ALF package^15^ with the nonlinear loss function^17^ using the BLaDE module^22^ of the CHARMM molecular dynamics software package,^23^ and used the CHARMM36 force field for proteins.^24^ The relative unfolding free energy estimates were calculated by comparing the free energy differences between the alchemical transformations in the unfolded ensemble and the folded ensemble (Supporting Information Figure S1). A new end point bias term differing from previous studies was introduced to better fit the alchemical barriers because the old end point bias broke down for the larger number of substituents in this study (Supporting Information Equation S3). Simulations included 22 mutations due to the three protonation states of histidine. Histidine free energies were calculated using the previously described method of obtaining the reference energy and performing a Boltzmann average over the three protonation states.^17^ Each site utilized 5 independent trials (with 5 replicas per trial^14^) to calculate uncertainties from bootstrapping over trials. The five independent trials were carried out for the folded and unfolded ensembles for a total of 1.5 μs of sampling for all folded ensembles and 1.7 μs for all unfolded ensembles.

**Figure 1:**
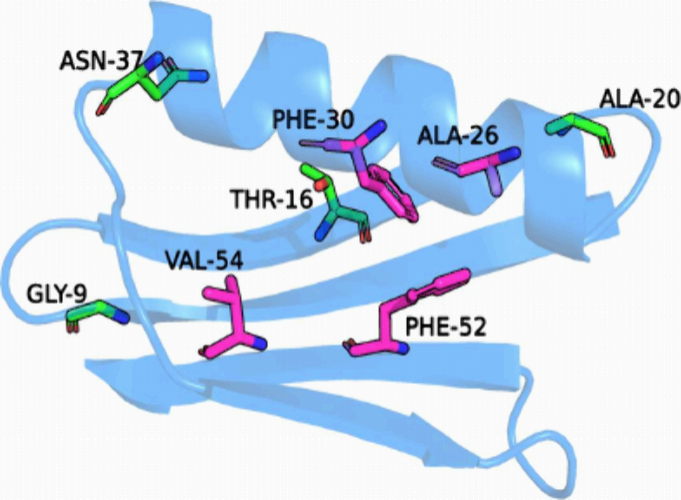
B1 domain of Protein G. The 4 selected surface mutation sites are represented in green, and the 4 selected core sites are represented in pink.

The accuracy of computational methods is important and should correlate tightly with experimental measurements. In previous large mutagenesis studies, nonalchemical methods such as Rosetta achieved Pearson correlation factors of up to 0.64 (with correlations of 0.53, 0.73, and 0.37 for surface, boundary, and core sites).^20^ Alchemical free energy calculations using FEP+ perform better and achieve Pearson correlations of 0.71 to 0.82 and root-mean-square errors (RMSE) of about 1.1 kcal/mol, though comparison is hindered by different data sets.^5,25^ In λ-dynamics simulations run using ALF, some mutations are too poorly sampled to estimate bootstrap uncertainties or sometimes to calculate free energy at all. These mutations are excluded from the accuracy calculations. To compare CS and TLF on equal footing, only the shared subset of mutations with enough bootstrapping samples to produce uncertainties in both CS and TLF calculations is used to evaluate accuracy.

At surface sites, CS and TLF with λ-dynamics both achieved higher accuracy than nonalchemical methods. Accuracy metrics relative to experiment were computed by aggregating the shared subset of mutations sampled by both methods across all four surface sites into a single set. In this shared subset of surface mutants, λ-dynamics achieved high Pearson correlation with experiment of 0.84 for CS and 0.82 for TLF. The RMSEs to experimental values for the shared subset of surface mutants were 0.89 and 0.92 kcal/mol for CS and TLF respectively (Figure 2 and Table 1). At these well-behaved surface sites, CS slightly improves correlation of the calculated values relative to TLF, though the slight improvements by CS are not significant. Proline and aspartic acid were the most difficult to sample for CS because they were the most destabilizing mutations experimentally, and were omitted from analysis for some sites as described above. For most mutants, the convergence and sampling of the folded ensembles are similar between CS and TLF, meaning the biases transferred from the unfolded ensemble in CS are sufficient to visit most or all substituents.

**Figure 2:**
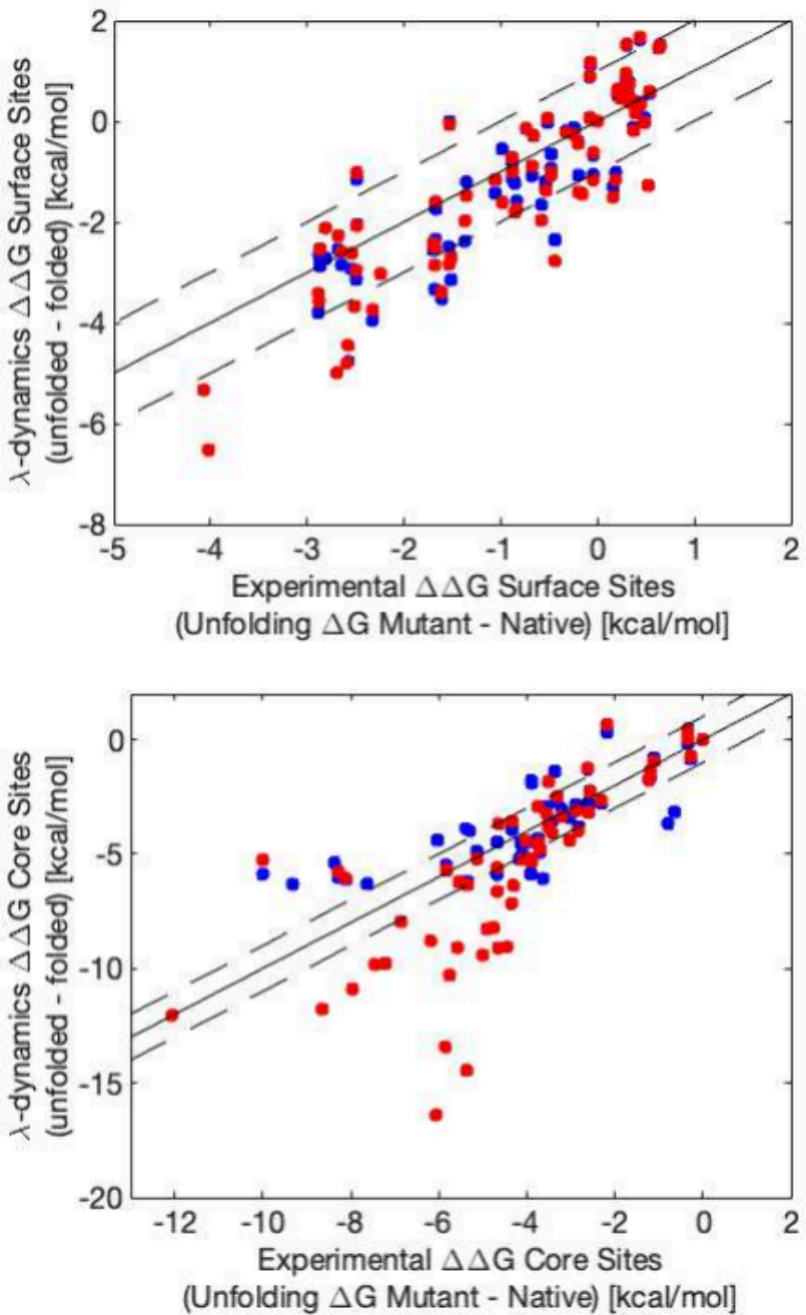
Comparison of correlation between competitive screening λ-dynamics and traditional landscape flattening λ-dynamics against experimentally determined relative unfolding free energies in the 4 surface sites (top) and the 4 core sites (bottom). TLF measurements are in red, and CS measurements are in blue. The solid black line represents *y* = *x*, and the dashed lines represent *y* = *x* ± 1.

**Table 1:** Comparison of CS and TLF with Experiment in Protein G within the Common Subset of Mutations Sampled by Both Methods^a^

Surface					
	CS		TLF		
Mutation Site	RMSE (kcal/mol)	R	RMSE (kcal/mol)	R	N common
G09	1.19	0.71	1.24	0.74	20
T16	0.86	0.83	0.92	0.84	18
A20	0.80	0.73	0.71	0.73	18
N37	0.57	0.77	0.60	0.77	18

Core					
	CS		TLF		
Mutation Site	RMSE (kcal/mol)	R	RMSE (kcal/mol)	R	N common
A26	0.65	0.97	1.09	0.97	9
F30	1.58	0.81	1.69	0.79	15
F52	1.95	0.71			0
V54	0.85	0.86	0.90	0.84	14

a N represents the number of mutants sampled by both methods.

In contrast to surface sites, in the core sites with many energetically unfavorable mutations, CS outperforms TLF in accurately reproducing experimental values. While CS and TLF sampled most mutants in the surface sites, at core sites CS sampled fewer of the 20 amino acids included in each simulation. However, this is the intended purpose of CS: to focus sampling on the most energetically favorable mutations and avoid sampling unfavorable mutations. This was successfully accomplished as all mutations that were unsampled during the CS simulations had folding free energy penalties sufficient to unfold the protein of over −4 kcal/mol.

More importantly, the focused sampling enabled CS to achieve lower RMSE and higher correlation to experiment than TLF for the shared subset of mutations that were sampled by both methods (Figure 2 and Table 1). CS outperformed TLF in core sites with a RMSE of 1.14 kcal/mol and a correlation of 0.86 compared to TLF which had a RMSE of 1.31 kcal/mol and a correlation of 0.82 for the set of all mutants sampled by both methods. Accuracy differences were even more striking outside the shared subset where CS obtained a RMSE of 1.43 kcal/mol and correlation of 0.81 while TLF had a RMSE of 2.84 kcal/mol and correlation of 0.76 (see Supporting Information). Generally, TLF had a tendency to overestimate the impact of unfavorable mutations, with overestimates reaching up to −7 kcal/mol. This trend was seen across all core sites on the subset of residues that were sampled by both CS and TLF. The superior performance of CS arises because CS focuses more sampling on the favorable mutations, while TLF unsuccessfully attempts to equally sample the entire mutation ensemble.

An additional advantage of CS over TLF is the improved stability and robustness of simulations. Several alchemical sampling problems were seen in TLF simulations such as overestimation of the effect of unfavorable mutations in all core sites. Conformational sampling problems were encountered with TLF, because the deleterious mutations disrupt the folded ensemble experienced by remaining mutants. TLF also exhibited numerical stability issues; in one site, F52, TLF was unable to estimate free energies at all because the simulation of the folded ensemble would crash upon starting production. The increased stability of CS simulations stems from improved sampling of alchemical space, resulting in improved conformational sampling and numerical stability.

Improvements in alchemical sampling that arise from elimination of sampling unfavorable mutations can be quantified by increased alchemical transitions between mutations in CS versus TLF. CS improved the number of alchemical transitions by up to 1 order of magnitude in both core and surface sites versus TLF (Figure 3). Furthermore, the number of transitions by CS correlates with stability, indicating CS gives better convergence for the most favorable mutations (Figure 3). This increased convergence is a byproduct of the focus on sampling favorable mutations to the ensemble, which prevents the heightened disruption of the conformational space caused by the intermolecular interactions of unfavorable mutations.

**Figure 3:**
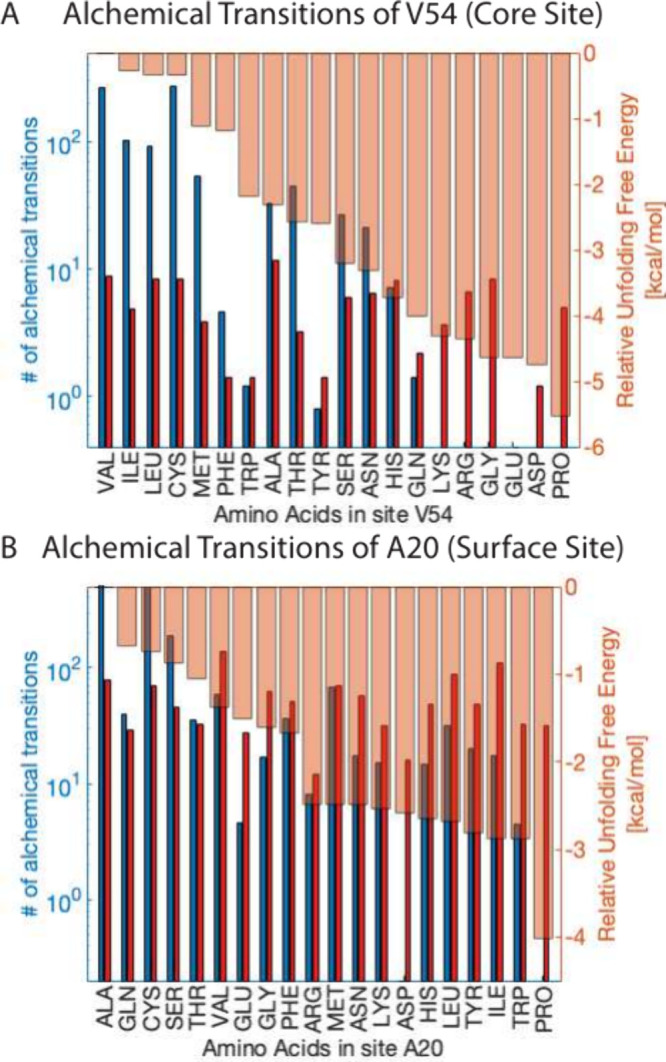
Comparison of the number of alchemical transitions per mutant in a simulation. CS transitions are shown in blue and TLF in red. (A) is V54, a core site with many unfavorable mutants and (B) is A20, a surface site. Many transitions are required for converged free energy estimates. Significantly more transitions are seen in core sites using CS compared to TLF, and those transitions are focused on the most stable mutations.

Improvements in conformational sampling can be quantified by root-mean-square difference (RMSD) from the starting structure, which were obtained via the MDanalysis software package.^26,27^ CS simulations retained the starting protein structure better than TLF, with RMSD of the backbone remaining up to 0.8 Å lower (Figure 4). Because TLF samples all mutants equally, it drives sampling of unfavorable mutations that disrupt the conformation of the protein and begin protein unfolding. Because simulations are far too short to sample partial unfolding reversibly, favorable mutations that favor the native conformation sample less well in the disrupted ensemble, and unfavorable mutations also sample poorly, leading to large inaccuracies. The transferred biases in CS instead favor sampling mutations with free energies more favorable than the reference ensemble, effectively preventing the appearance of unfavorable mutations that would disrupt the folded ensemble.

**Figure 4:**
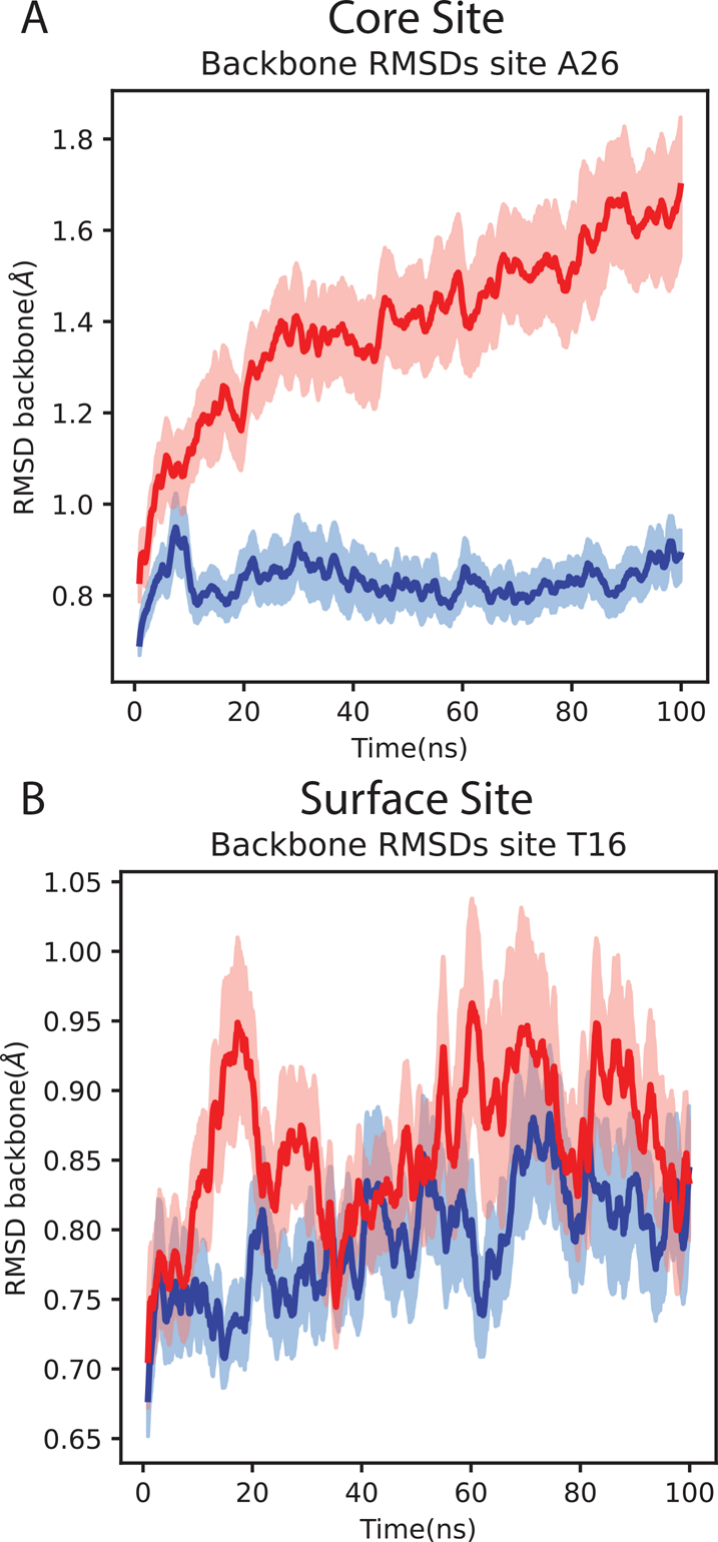
Comparison of the RMSD of the protein backbone between CS and TLF. (A) is A26, a core site with many unfavorable mutants and (B) is T16, a surface site. CS is shown in blue, and TLF is shown in red. The standard error of the mean of each method is shown in the lightly shaded portions of each respective color. CS disrupts the folded structure less than TLF for core sites, leading to improved accuracy.

The core strength of CS is the ability to focus sampling on the most favorable substitutions, so CS is useful beyond sampling all 20 amino acids at a site. Similar methods to CS have been used in constant pH molecular dynamics to calibrate the free energy calculations of protonation at specific pH values through obtaining free energy values of a reference state.^28^ CS could also be useful in drug design to transfer biases from the reference ensemble of the ligand in solvent to the target ensemble of the protein–ligand complex. In drug design this could sort large groups of ligands using a more accurate free-energy based method than docking while being more efficient than previous λ-dynamics methods such as TLF.

We expect that CS should provide the same benefits of improved stability and preferential sampling when applied to larger physical systems such as bigger proteins, albeit with the increased computational cost inherent with simulating larger molecular systems. CS also reduces the computational cost of evaluating thermodynamic stability compared to TLF, because no sampling is needed to flatten the biases in the folded ensemble.

This work has investigated how λ-dynamics is able to effectively evaluate the free energy differences of all 20 amino acids using the CS (competitive screening) method in the B1 domain of Protein G, a well-characterized experimental system. Unfolding free energy calculations were performed on all 20 amino acids at 4 well-behaved surface sites and 4 difficult core sites to compare CS with TLF (traditional landscape flattening). CS was able to more accurately evaluate mutations in difficult sites, with better correlation to experiment and low RMSE, while TLF generated free energies for more mutations per site that had poorer accuracy and RMSE. Consequently, CS is more effective in design studies identifying optimal mutants while TLF is more effective in studies evaluating all mutants. CS successfully filtered out the most energetically unfavorable mutations, as intended, since the mutations remaining unsampled in the folded simulation corresponded to those that were most detrimental to protein folding experimentally. CS also improved convergence and sampling of the folded ensemble simulations, which can be seen through increased alchemical transitions of the most favorable mutants and the lowered backbone RMSD during simulation compared to TLF, as unfavorable mutations disrupt the folded ensemble. This work demonstrates that λ-dynamics can obtain accurate free energy measurements from mutation sites with many deleterious mutations in proteins and can efficiently explore large portions of the chemical space while accurately classifying energetic favorability of substituents. This enables rapid discernment of thermodynamic stability of mutations and filtering of unfavorable mutations, which could help guide experimental protein engineering.

## Data Availability

Full results, example run input files, and scripts used for landscape flattening are available for download at https://github.com/RyanLeeHayes/PublicationScripts/blob/main/2025CompetitiveScreening.tgz

## Supporting Information Available

Free energy diagrams, alchemical bias potential details, simulation details, results by site, and a FoldX comparison^29^ (PDF)
