# Supplementary material for: Predicting Thermodynamic Stability at Protein G Sites with Deleterious Mutations Using λ-Dynamics with Competitive Screening
Source: J Phys Chem Lett. 2025 Mar 21;16(13):3206–11. doi: 10.1021/acs.jpclett.5c00260 (PMC11973915; doi:10.1021/acs.jpclett.5c00260)
Supplement: Supplementary file 1 — jz5c00260_si_001.pdf [file jz5c00260_si_001.pdf]

Predicting thermodynamic stability at Protein G sites with deleterious mutations using λ-dynamics with competitive screening

Christopher Yeh[†] and Ryan L. Hayes[*,‡,†]

†*Department of Pharmaceutical Sciences, University of California Irvine*

‡*Department of Chemical and Biomolecular Engineering, University of California Irvine*

E-mail: rhayes1@uci.edu

Free Energy Diagram

The main text discusses the use of relative free energy calculations to determine the thermodynamic stability of mutations. Figure S1 shows the alchemical cycle for performing a free energy calculation to compare two mutations at the same residue. The physical processes would measure the folding free energy difference for a single sequence by transforming the unfolded ensemble to the folded ensemble through conformational transformation; one then takes the difference between the folding free energy for each sequence. The alchemical processes transform between sequences of the compared mutations in either the unfolded or folded ensembles; the mutational free energies for each ensemble are then subtracted from one another. For the simulations in Protein G, the calculated free energy differences are between the native residue on one arm of the alchemical cycle and a mutant on the other. The relative free energies of the experimental results were found from the difference between the unfolded free energy of the native sequence and the mutated sequence in the experimental dataset of Protein G from Tsuboyama et al.[1]

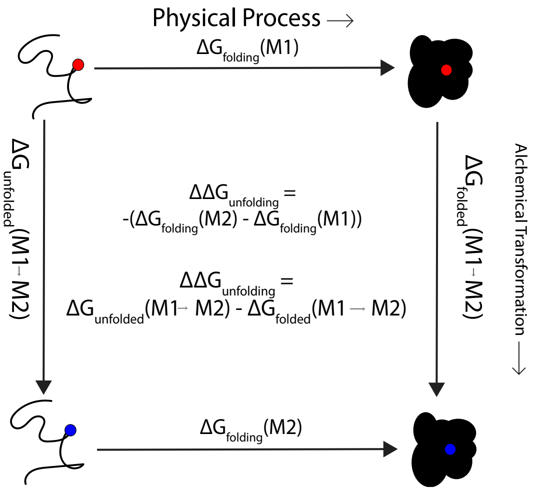

Figure S1: The thermodynamic cycle for computing unfolding free energies($\Delta\Delta G_{\text{unfolding}}$). The physical process of folding and unfolding would occur in the horizontal direction of the cycle, where the unfolded ensemble transforms into the folded ensemble.The alchemical process is depicted as the vertical arms of the cycle and would convert one sequence(mutated residue shown in red) to another sequence(mutated residue shown in blue). The $\Delta\Delta G_{\text{unfolding}}$ is most efficiently obtained through the comparison of the two rapidly converged alchemical processes instead of the slowly converging physical processes.

Bias Calculation

In previous papers on λ-dynamics, energetic endpoint traps were discovered near the value of $\lambda = 0$ with a very narrow width at half maximum depth of $a = 0.017$, when transforming into another substituent. These endpoint traps required an additional bias potential to flatten the energetic penalty, which is given by

$$U_\omega = \sum_s^M \sum_i^{N_s} \sum_{j \neq i}^{N_s} \omega_{si,sj} \frac{\lambda_{si}}{\lambda_{si} + a} \lambda_{sj} \tag{S1}$$

where $a = 0.017$, the sum on s runs over all sites M, the sum on i runs over all N_s substituents at site s, and the sum on j runs over all substituents at site s other than i. This bias is required to overcome the energetic penalty for creating the solvent cavity necessary to make way for the newly appearing substituent i near $\lambda_{si} = 0$.

However, the bias coefficients $\omega_{si,sj}$ determined by ALF, which correspond to the energy required to make way for substituent i when substituent j is near $\lambda_{sj} = 1$ were inconsistent between pairwise simulations and the simulations of the whole ensemble. Furthermore, while pairwise simulations with ALF always converge to the same $\omega_{si,sj}$ values, simulations with all 22 sequences differ substantially. This is due to the unintended effect that if $\omega_{2,1}$ pays the penalty to make a cavity to raise λ_2 above α, then a large portion of the energy $\omega_{3,1}$ to make a cavity to also raise λ_3 above α has already been paid. To rectify this issue, a new bias potential for the endpoint bias of the form

$$U_\omega = \sum_s^M \sum_i^{N_s} \sum_{j \neq i}^{N_s} \omega_{si,sj} \frac{\lambda_{sj}}{a + (1 - \lambda_{sj})} \lambda_{si} \tag{S2}$$

is developed, which is identical in pairwise simulations where $\lambda_{si} + \lambda_{sj} = 1$, but different when more substituents are present. This bias can be computed using the same code by refactoring it as

$$U_\omega = \sum_s^M \sum_i^{N_s} \sum_{j \neq i}^{N_s} -\omega_{si,sj} \frac{\lambda_{sj}}{\lambda_{sj} + (-1 - a)} \lambda_{si} \tag{S3}$$

Simulation Details

In addition to the simulation details mentioned in the main text, several more steps were applied to the ensembles before simulation. The unfolded ensembles were subjected to 50 steps of steepest descent and 50 steps of adopted basis Newton Raphson. The unfolded ensembles were then equilibrated for 0.125 ns using the LEAP integrator using the BLaDE module in CHARMM. CHARMM-GUI scripts were used to generate the solvent box and to equilibrate structures. For all flattening runs and production runs using ALF, force switching was used for van der Waals interactions with a switching radius of 9 Å and a cutoff radius of 10 Å.[2,3] PME electrostatics were used for long-range electrostatics by scaling charges by λ and using a cutoff of 10 Å, an interpolation order of 6, $\kappa = 0.32$ Å^{-1}, and a grid spacing of approximately 1.0 Å.[4] Simulations were run with a time step of 2 fs, a Langevin thermostat, and a Monte Carlo barostat. Simulations used the CHARMM36 force field for proteins.[5] and the TIP3P force field for water.[6]

Stability Scatterplots and Tables by Site

λ-dynamics achieved high Pearson correlation over the set of all surface site mutants with 0.84 for CS and 0.82 for TLF. The RMSE to experimental values were 0.89 and 0.92 kcal/mol for CS and TLF respectively. At core sites, CS outperformed TLF on the common subset of mutations sampled by both methods, but the difference in performance is even greater when considering mutations beyond the common subset. CS outperformed TLF in RMSE and correlation for all mutations sampled by their respective methods, with the RMSE for CS for all core site mutants being 1.43 kcal/mol compared to 2.84 kcal/mol for TLF and a Pearson correlation of 0.81 versus 0.76.

Table S1: Comparison of CS and TLF with experiment in Protein G for all mutations sampled under each respective method

Surface All						
Mutation	CS			TLF		
Site	RMSE (kcal/mol)	R	N simulated	RMSE (kcal/mol)	R	N simulated
G09	1.19	0.71	20	1.24	0.74	20
T16	0.86	0.83	18	1.03	0.92	20
A20	0.80	0.73	18	1.01	0.77	20
N37	0.57	0.77	18	0.69	0.95	20
**Core All**						
Mutation	CS			TLF		
Site	RMSE (kcal/mol)	R	N simulated	RMSE (kcal/mol)	R	N simulated
A26	0.65	0.97	9	2.99	0.86	20
F30	1.58	0.81	15	3.51	0.60	20
F52	1.95	0.71	15			0
V54	0.80	0.87	14	1.68	0.87	20

Table S2: Comparison of CS and TLF with experiment in Protein G within the common subset of mutations sampled by both methods

Surface Common					
Mutation	CS		TLF		
Site	RMSE (kcal/mol)	R	RMSE (kcal/mol)	R	N common
G09	1.19	0.71	1.24	0.74	20
T16	0.86	0.83	0.92	0.84	18
A20	0.80	0.73	0.71	0.73	18
N37	0.57	0.77	0.60	0.77	18
**Core Common**					
Mutation	CS		TLF		
Site	RMSE (kcal/mol)	R	RMSE (kcal/mol)	R	N common
A26	0.65	0.97	1.09	0.97	9
F30	1.58	0.81	1.69	0.79	15
F52	1.95	0.71			0
V54	0.85	0.86	0.90	0.84	14

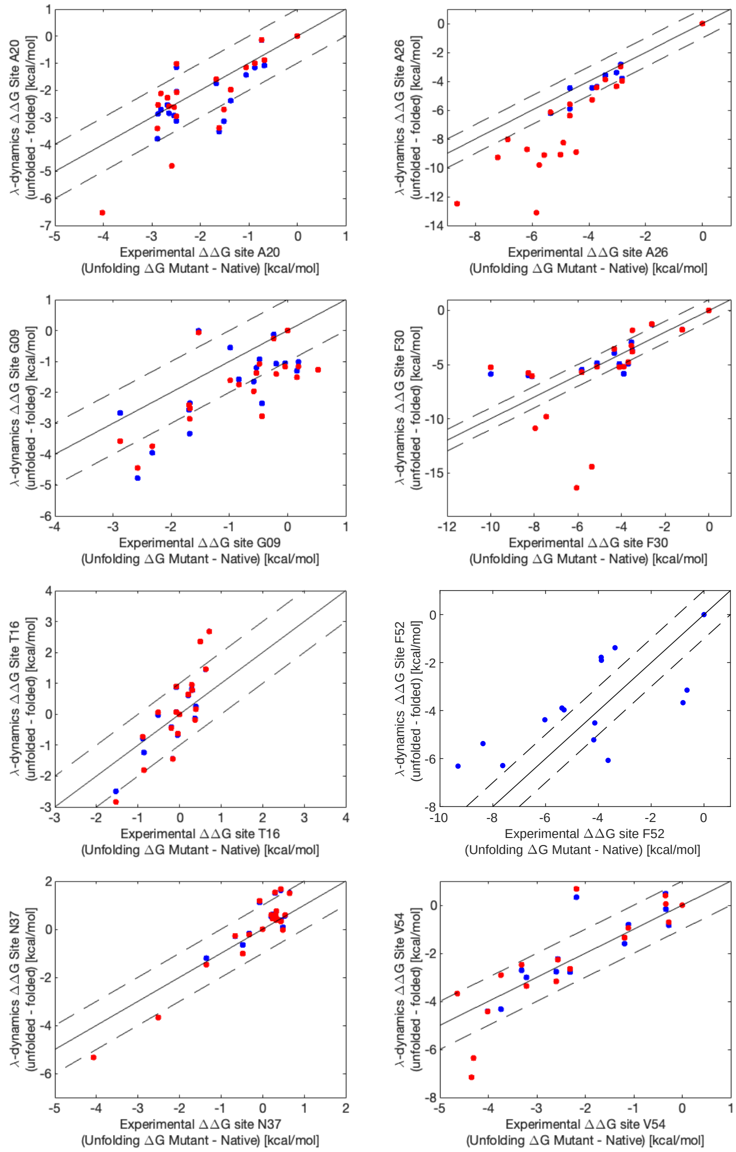

Figure S2: Correlation between λ-dynamics results and experimental results for unfolding free energies for all 20 amino acids in all sites shown. CS is represented in blue while TLF is represented in red. The region of ± 1 kcal/mol is shown by the dotted lines while the solid black line is y=x.

Alchemical Transitions by Site

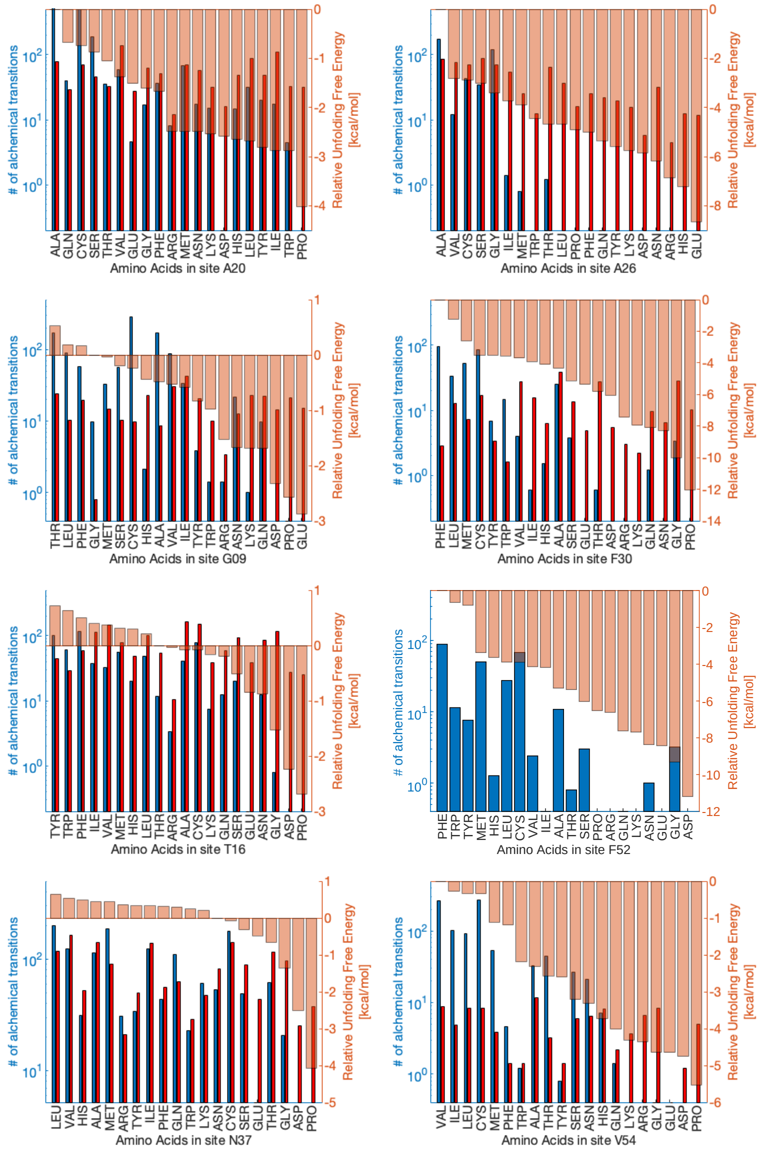

Figure S3: Comparison of the number of alchemical transitions per mutant in a simulation, with the 4 surface sites on the left and 4 core sites on the right. Competitive screening transitions are shown in blue and traditional landscape flattening in red. Many transitions are required for converged free energy estimates. Significantly more transitions are seen in core sites using CS compared to TLF, and those transitions are focused on the most stable mutations.

RMSDs by Site

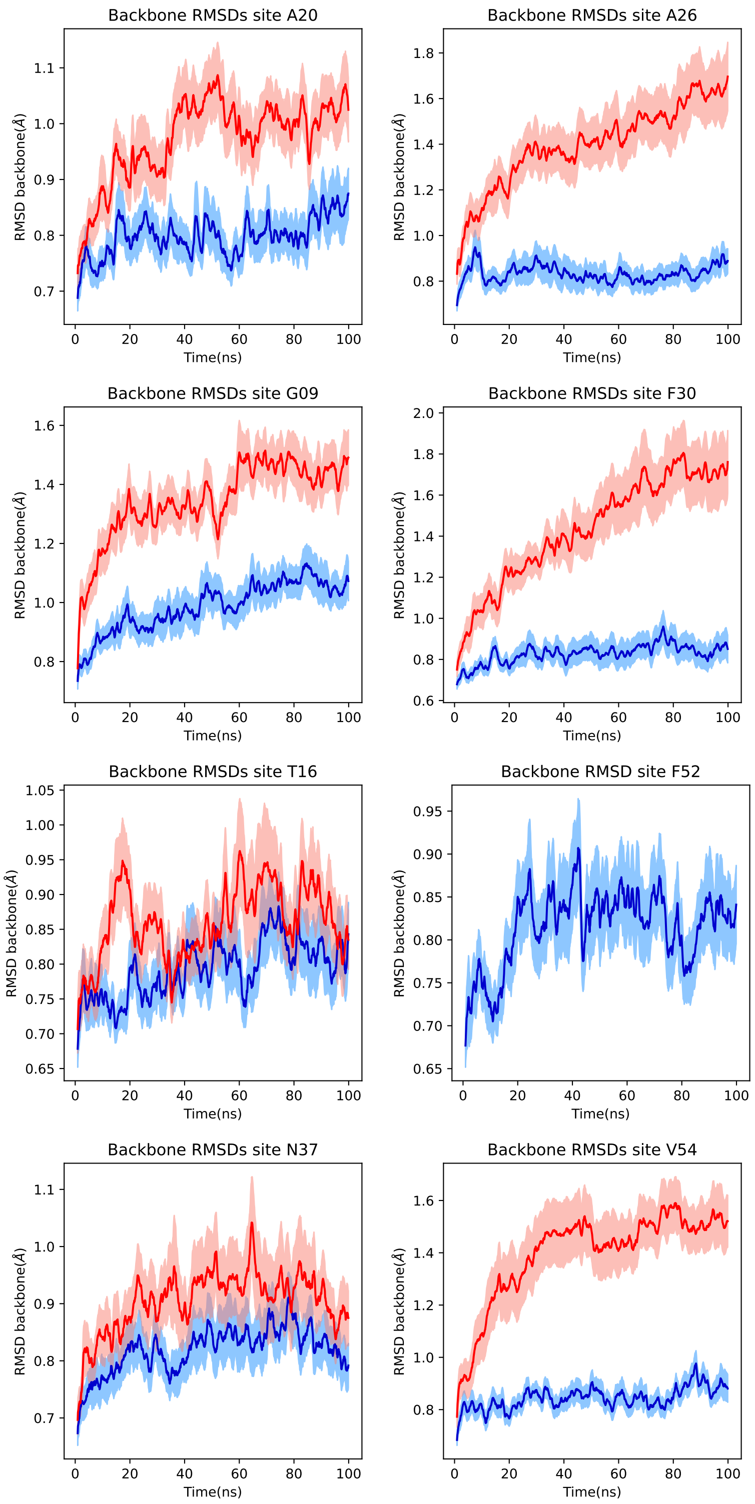

Figure S4: Comparison of the backbone RMSDs for competitive screening and traditional landscape flattening across all 4 surface sites and 4 core sites on the left and right respectively. CS is represented in blue and TLF in red. The standard error of the mean of each method is shown in the lightly shaded portions of each respective color. CS disrupts the folded structure less than TLF for core sites, leading to improved accuracy.

FoldX Calculations

We have studied the behavior and calculations of FoldX,[7] and included a reference set of calculations in the raw data posted online. The behavior of FoldX varies widely between sites, because some protein G sites are part of FoldX training data.[7] Sites that have been augmented with experimental data of point mutants of Protein G perform well with as low as 1.8 kcal/mol RMSE, and have good correlation up to 0.8, but other sites have large RMSEs of 6 kcal/mol and 0.1 correlation factors. In contrast, λ-dynamics demonstrates consistently accurate prediction of unfolding energies across all sites. The FoldX predictions of some core sites were able to predict correlations similar to CS, but those sites had experimental data added to the FoldX force field to adjust parameters during better quality estimation from the dataset provided from Guerois et al. 2002,[7] while CS and TLF rely solely on the force field and alchemical molecular dynamics to provide free energy insight. Even so, FoldX has a higher RMSE and lower correlation in all sites compared to both λ-dynamics approaches.

Raw Mutation Free Energies

Github repository contains spreadsheets of free energies for the unfolded ensemble, TLF folded ensemble, CS folded ensemble, and results from FoldX calculations on the same set.[7] Repository also contains prep directories used to run ALF on each system, bash scripts to run TLF and CS, and a modified version of the ALF python package that uses the new biases described by Equation S2. The repository is available for download at `https://github.com/RyanLeeHayes/PublicationScripts/blob/main/2025CompetitiveScreening.tgz`.

References

(1) Tsuboyama, K.; Dauparas, J.; Chen, J.; Laine, E.; Mohseni Behbahani, Y.; Weinstein, J. J.; Mangan, N. M.; Ovchinnikov, S.; Rocklin, G. J. Mega-scale experimental

analysis of protein folding stability in biology and design. *Nature* **2023**, *620*, 434–444.

(2) Lee, J.; Cheng, X.; Jo, S.; MacKerell, A. D.; Klauda, J. B.; Im, W. CHARMM-GUI input generator for NAMD, GROMACS, AMBER, OpenMM, and CHARMM/OpenMM simulations using the CHARMM36 additive force field. *Biophysical journal* **2016**, *110*, 641a.

(3) Steinbach, P. J.; Brooks, B. R. New spherical-cutoff methods for long-range forces in macromolecular simulation. *Journal of computational chemistry* **1994**, *15*, 667–683.

(4) Darden, T.; York, D.; Pedersen, L. Particle mesh Ewald: An N·log (N) method for Ewald sums in large systems. *The Journal of chemical physics* **1993**, *98*, 10089–10092.

(5) Best, R. B.; Zhu, X.; Shim, J.; Lopes, P. E.; Mittal, J.; Feig, M.; MacKerell Jr, A. D. Optimization of the additive CHARMM all-atom protein force field targeting improved sampling of the backbone ϕ, ψ and side-chain $\chi 1$ and $\chi 2$ dihedral angles. *Journal of chemical theory and computation* **2012**, *8*, 3257–3273.

(6) Jorgensen, W. L.; Chandrasekhar, J.; Madura, J. D.; Impey, R. W.; Klein, M. L. Comparison of simple potential functions for simulating liquid water. *The Journal of chemical physics* **1983**, *79*, 926–935.

(7) Guerois, R.; Nielsen, J. E.; Serrano, L. Predicting Changes in the Stability of Proteins and Protein Complexes: A Study of More Than 1000 Mutations. *Journal of Molecular Biology* **2002**, *320*, 369–387.
